# Eco-friendly, sensitive and inventive first derivative synchronous spectrofluorimetric determination of ciprofloxacin and phenylephrine in their pure form, single and combined eye drops

**DOI:** 10.1186/s13065-024-01131-4

**Published:** 2024-02-05

**Authors:** Neamat T. Barakat, Amina M. El-Brashy, Mona E. Fathy

**Affiliations:** Department of Pharmaceutical Analytical Chemistry, Faculty of Pharmacy, Mansoura University, Mansoura, 35516 Egypt

**Keywords:** Ciprofloxacin hydrochloride, Phenylephrine hydrochloride, Derivative spectrofluorimetriy, Eye drops, Greenness assessment

## Abstract

A facile, sensitive, accurate and green spectrofluorimetric method was evolved for the assay of ciprofloxacin hydrochloride (CFX) and phenylephrine hydrochloride (PLN) in their co-formulated eye drops with their challengeable ratio of 30:1 for CFX and PLN, respectively. Such drops are clinically used for the treatment of eye bacterial infections. They relieve the symptoms of infection by stopping further growth of the causative microorganisms. The assay principle based on first-order synchronous spectrofluorometric scan using Δ λ = 40 nm in which PLN peak amplitudes were recorded at 283.4 nm, meanwhile CFX was measured at 326.2 nm in the same scans. The calibration curves were linear within the concentration ranges: 0.02–0.5 µg/mL and 0.5–5.0 µg/mL for PLN and CFX, respectively. The method validation was confirmed following the International Conference of Harmonization (ICH) Guidelines. This suggested method was the first one that described simultaneous analysis of PLN and CFX by a spectrofluorimetric technique. In this method, green analytical procedures were implemented to lessen occupational and environmental perils and method greenness was assessed by four assessment tools. GAPI, NEMI, AGREE and Analytical eco-scale were applied to this study and it was deduced from their results that the method had high degree of greenness as it fulfilled all requirements of GAPI, NEMI pictograms and it had high scores of analytical eco scale (97) and AGREE methods (0.82). Subsequently, it was successfully applied to assay both drugs in pure forms, pharmaceutical single and co-formulated eye drops.

## Introduction

Synchronous fluorimetric technique attracts great attention in pharmaceutical analysis because of its simplicity and sensitivity. It also allows scanning of the excitation and emission wavelengths simultaneously [1]. It is more advantageous than conventional fluorimetric approach due to its higher selectivity, high speed, spectral simplicity and less light scattering interference [2]. These merits authorize this approach to be applied in the resolution of mixtures with overlapped spectra and in achieving data for quantitative determination [2]. Moreover, it is more sensitive because the amplitude of the derivative signal is inversely proportional to the original spectrum band width [2]. So, the synchronous fluorescence spectroscopy combined with derivative amplitude has been favored in resolving multi-component mixtures and analyzing substances in presence of interferences or contaminants [3]. Furthermore, introduction of greenness assessment methods to the entire analytical process grantees that the method has good ecological impact and not cause any harmful effects to human or environment. So, four assessment tools were applied to evaluate this study.

**Fig. 1:**
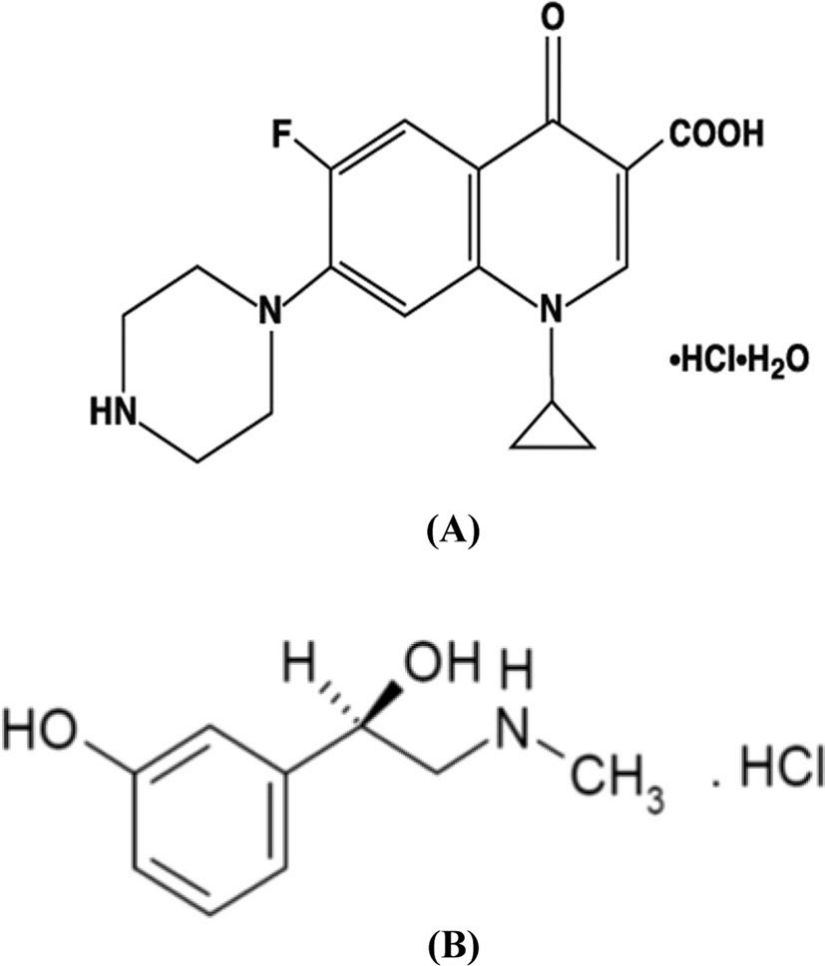
Chemical structure of **A** CFX and **B** PLN

Ciprofloxacin hydrochloride (CFX); is the mono hydrochloride monohydrate salt of 1-cyclopropyl-6-fluoro-4-oxo-7-piperazin-1-ylquinoline-3-carboxylic acid [4] (Fig. 1A). It is a second generation fluorinated quinolone bactericidal. It is used for treatment of bacterial infection through inhibiting essential enzymes in the reproduction of bacterial DNA as DNA gyrase and topoisomerase IV. It has a broader spectrum of activity and greater potency in vitro than non-fluorinated quinolone [5].

Several analytical techniques have been evolved for analyzing CFX as; some of analytical methods were summarized in the reported review [6], spectrophotometry [7–12], spectrofluorimetry [13, 14], electrochemistry [15–17], high-performance liquid chromatography [18–20], capillary electrophoresis [21] and titration [22].

Phenylephrine hydrochloride (PLN); is benzenemethanol,3-hydroxy-α [(methyl amino) methyl]-hydrochloride (R) [4]. (Fig. 1B). It is a sympathomimetic and it has direct effects on adrenergic receptors. It is designated to be nasal decongestant, mydriatic and hypertensive agent in acute hypotensive state [5].

Literature survey shows that PLN was assayed by several techniques such as; spectrophotometry [23–28], spectrofluorimetry [29], high-performance liquid chromatography [30–32] and capillary electrophoresis [33].

Both drugs are official in the British Pharmacopoeia (BP) [34] and in the United States Pharmacopeia [35]. By reviewing the literature, it turns out that there were spectrophotometric [36] and high-performance liquid chromatographic [37] studies had been investigated and no spectrofluorimetric method was reported for concurrent analysis of both drugs.

It is the first time for simaltenious assay of CFX and PLN through application of synchronous fluorescence approach without need to high cost procedures or advanced methods. It does not require any tedious and time consuming pretreatment steps. Moreover, the proposed method is simple, sensitive, valid and it fulfills all criteria of greenness, which make it more favorable to be applied in quality control of the suggested drugs.

## Experimental

### Devices

The whole work was performed using Cary Eclipse fluorescence spectrophotometer with a xenon lamp. A high voltage of 800 V was applied with 15 smoothing factor and 5 nm slit width. The derivative spectra were obtained using a filter size of 20.0 nm (USA), the pH meter was used to adjust pH of the prepared buffers (Consort, P-901, Belgium) and ultrasonic bath model SS-101H, 230 (USA).

### Solvents and materials

HPLC grade solvents and analytical reagent grade chemicals were used as;

Acetonitrile, methanol and ethanol were brought from Sigma Aldrich (Germany). While, β-cyclodextrin, tween-80, sodium dodecyl sulfate (SDS), cetrimide, sodium hydroxide, boric acid, acetic acid, phosphoric acid, sulfuric acid and hydrochloric acid were obtained from El Nasr Chemical Co., (Egypt).

Ciprofloxacin HCl pure sample was obtained from the National Organization for Drug Control and Research (NODCAR), (Cairo, Egypt) with a purity of 99.81 ± 1.73% as calculated by comparison method [36]. While, Phenylephrine HCl pure powder was kindly obtained from Global Advanced Pharmaceuticals (6th October city, Giza, Egypt) with purity of 99.93 ± 1.62% as calculated by comparison method [36].

### Pharmaceutical dosage forms

**Ciprocin® 0.3% eye drops**; (contain 3 mg/mL Ciprofloxacin hydrochloride) were produced by: Egyptian International Pharmaceutical Industries Co., (10th Ramadan city, Egypt) and **Phenylmedrin® 2.5% eye drops;** (contain 25 mg/mL Phenylephrine hydrochloride) were manufactured by Monopharma for pharmaceutical industries, (Badr City, Cairo, Egypt).

### Buffer solutions

0.2 M aqueous solutions of Britton Robinson buffer covering the pH range 2.0–12.0, borate buffer of pH range 6.0–10.0 [35] and acetate buffer with pH range 4.0–5.5 [35] were prepared.

### Standard solutions

200.0 μg/mL standard stock solutions of PLN or CFX were prepared separately in 100 mL volumetric flask by dissolving 20.0 mg from each drug in 100 mL distilled water. Subsequent dilutions were done as appropriate to obtain the working solutions using the same diluting solvent.

## Procedures

### Calibration curves

Working solutions within the concentration range of 0.02–0.5 µg/mL and 0.5–5.0 µg/mL for PLN and CFX, respectively were separately prepared in a set of 10 mL volumetric flasks. Then, the solutions were analyzed using synchronous fluorescence approach at Δ λ = 40 nm with scanning at a rate of 600 nm/min by 5 nm excitation and emission windows. Then, the first derivative synchronous spectra (^1^D) were obtained and peak amplitudes were measured at zero crossing point of each drug; 283.4 nm for PLN (zero crossing point of CFX) and 326.2 nm for CFX (zero crossing point of PLN). Calibration curves were then constructed by graphing the first derivative amplitudes (^1^D) versus each drug final concentrations to obtain corresponding regression equations.

### Assay of CFX / PLN in their synthetic mixtures

Different aliquots from standard solutions of CFX and PLN in their ratio of 30:1 were transferred to a set of 10 mL volumetric flasks. Then solutions were diluted with distilled water to the mark and mixed well. By applying steps of the procedure under "Calibration curves" Section, ^1^D amplitude values corresponding to each drug were measured concurrently and the corresponding concentrations were obtained from calibration curves or regression equations.

### Analysis of CFX/PLN in their commercial single eye drops

For analysis of CFX in Ciprocin® or PLN in Phenylmedrin® eye drops; Five bottles of each drug were evacuated and their contents were mixed well then certain volume equivalent to 10.0 mg of CFX or PLN was separately transferred into 100 mL volumetric flasks then diluted with distilled water to mark and mixed well. After that prepared solutions were diluted with the same diluting solvent to obtain desired working solutions. The procedure under "Calibration curves" Section was then followed and the content of CFX or PLN individual eye drops was obtained using the derived regression equations or referring to the corresponding calibration curves.

### Analysis of CFX/PLN in their prepared co-formulated eye drops

For the analysis of CFX and PLN in their prepared co-formulated eye drops, a volume of Phenylmedrin® eye drops equivalent to 10 mg PLN was accurately transferred to 100 ml measuring flask, then diluted with distilled water to the mark to prepare 100 µg/mL stock solution. After that, 4 mL from this solution was transferred to 100 mL measuring flask followed by a volume of Ciprocin® eye drops equivalent to 12 mg CFX and mixed well. This finally prepared solution was diluted to the mark with distilled water and mixed well to get CFX / PLN stock solution in their ratio of 30:1. Then, aliquots within the working concentration range were transferred into a set of 10 mL volumetric flasks and the suggested procedure under "Calibration curves" Section. was then performed. Finally, the content of co-formulated eye drops was accurately calculated by the derived regression equation or calibration curves.

## Results and discussion

**Fig. 2:**
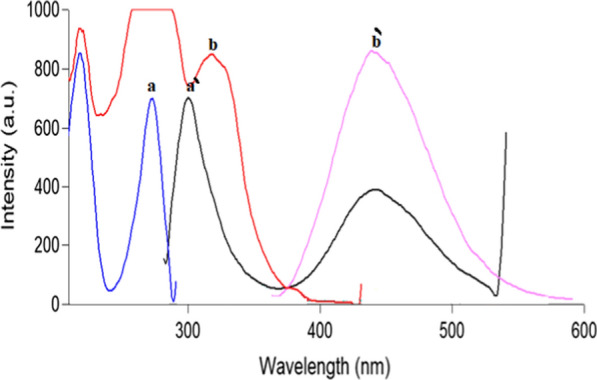
Excitation and emission fluorescence spectra of: (a, a') PLN (0.1 µg/mL), and (b, b') CFX (0.01 µg/mL), using water as a solvent

**Fig. 3:**
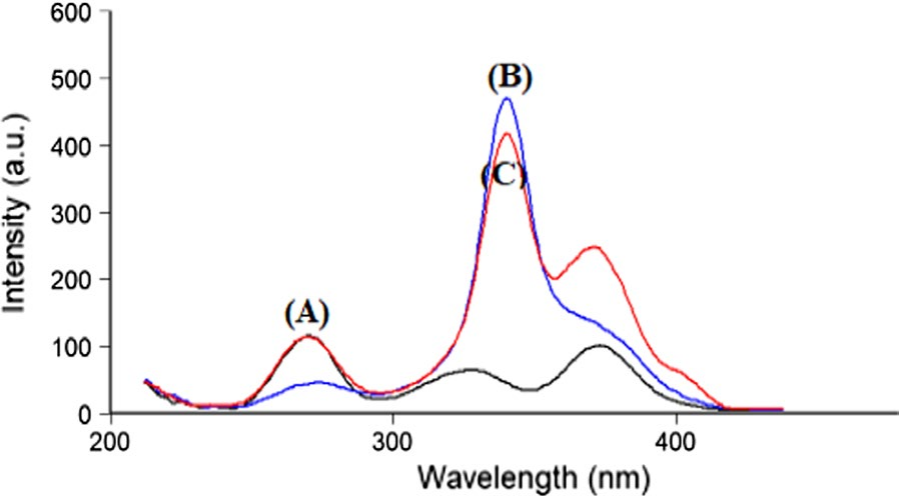
Synchronous spectra at Δ λ = 40 nm of: **A** PLN (0.05 µg/mL). **B** CFX (1.5 µg/mL). **C** A mixture of PLN / CFX (0.05 / 1.5 µg/mL)

**Fig. 4:**
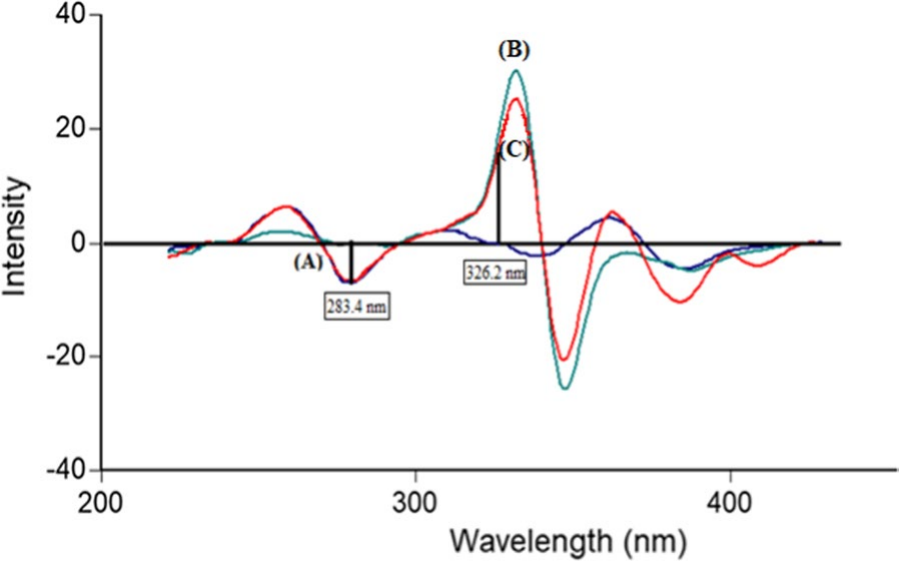
First derivative synchronous fluorescence spectra of: **A** PLN (0.05 µg/mL). **B** CFX (1.5 µg/mL). **C** Synthetic mixture of PLN/CFX (0.05 /1.5 µg/mL)

**Fig. 5:**
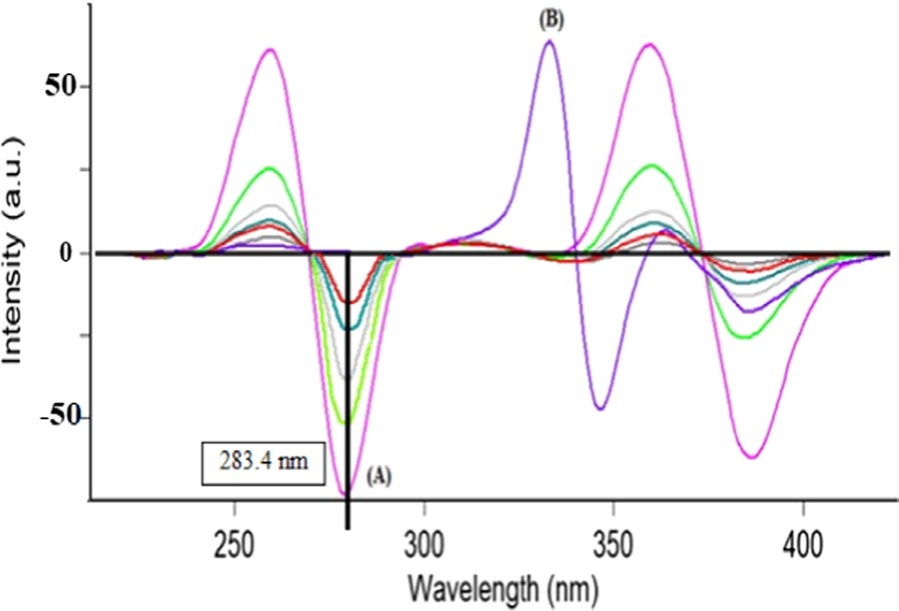
First derivative synchronous fluorescence spectra of: **A** PLN (0.02–0.05–0.07–0.1–0.2 and 0.5 µg/mL) at 283.4 nm. **B** CFX (4.0 µg /mL)

**Fig. 6:**
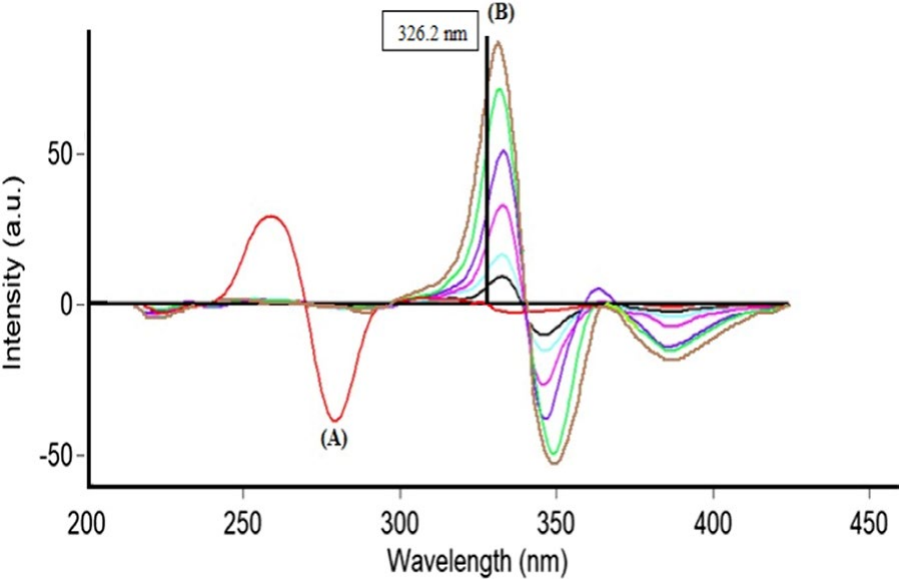
First derivative synchronous fluorescence spectra of: **A** PLN (0.5 µg/mL). **B** CFX (0.5–1.0–2.0–3.0–4.0 and 5.0 µg/mL).at 326.2 nm

PLN and CFX show intrinsic fluorescence at λ_ex_ 273 nm/λ_em_ 300 nm and λ_ex_ 318 nm/λ_em_ 446 nm for the two drugs, respectively as represented in (Fig. 2). Fluorescence spectra of both drugs were greatly overlapped which hindered simultaneous determination of CFX and PLN by conventional spectrofluorimetric methods. This problem is highly aggravated if it is wanted to assay both drugs in their combined dosage forms. To enhance the resolution of this mixture, synchronous scanning was conducted at Δ λ 40 but both drugs still could not be determined in presence of each other because of persistent peak overlap, as represented in (Fig. 3). So, ^1^D of synchronous spectra amplitudes of PLN and CFX were measured at 283.4 and 326.2 nm for the two drugs, respectively to improve the resolution and to increase the selectivity (Fig. 4). By this way, relied on the concept of zero-crossing point, both drugs could be assayed simultaneously with no need for the addition of any selective hazardous reagents or tedious pretreatment separation steps as shown in Figs. 5 and 6.

### Experimental parameters optimization

#### Optimal Δ λ selection

Δ λ value has a remarkable effect on the shape of the synchronous spectra, bandwidth and value of the signal. So a wide range of Δ λ (20–120 nm) was tried and it was found that Δ λ 40 nm was optimal one as at which maximum selectivity and highest synchronous fluorescence intensity were obtained.

#### Effect of different diluting solvents:

**Fig. 7:**
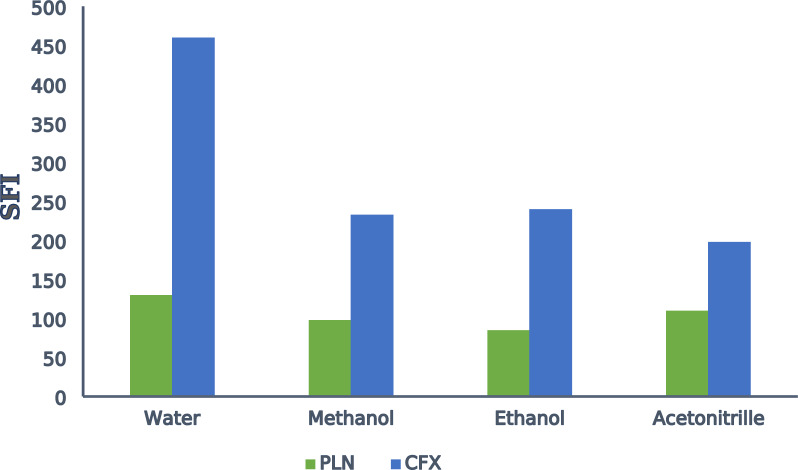
Effect of diluting solvents on relative synchronous fluorescence intensity for PLN (0.05 µg/mL) and CFX (1.5 µg/mL)

Various solvents as: distilled water, acetonitrile, ethanol and methanol were investigated. It was observed that the usage of distilled water gave the best sensitivity for both drugs (Fig. 7). So, distilled water was the diluting solvent of choice used through the whole study.

#### Effect of pH of the medium

It was evaluated utilizing 0.2 M Britton Robinson buffer with the pH range 2.0–12.0, 0.2 M borate buffer of the pH range 6.0–10.0, 0.2 M acetate buffer covering the pH range 4.0–5.5 in addition to 0.1 N HCl and 0.1 N NaOH. It was found that the change in the media pH had no positive or negative effect on the fluorescence intensity of both drugs. So, neutral pH of distilled water was applied during this work.

#### Effect of different organized media

**Fig. 8:**
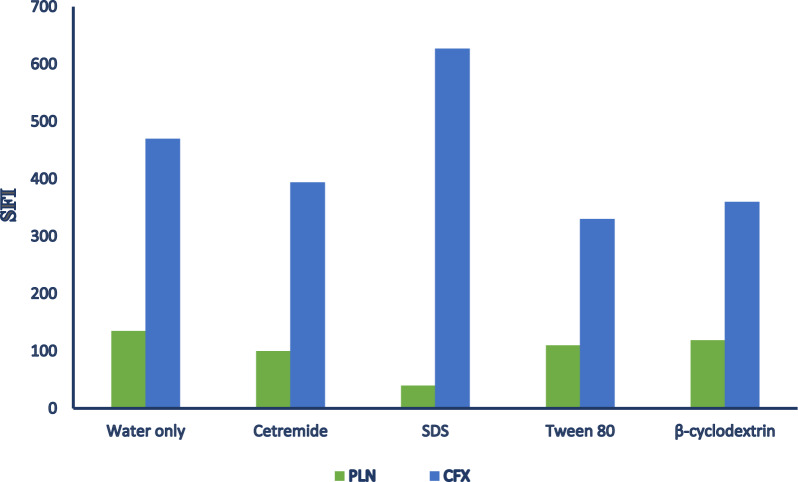
Effect of organized media on relative synchronous fluorescence intensity for PLN (0.05 µg/mL) and CFX (1.5 µg/mL)

For enhancing the suggested method sensitivity, various organized media were investigated; cetrimide, tween 80, β-cyclodextrin and SDS. It was observed that the use of these organized media caused decrease in intensity of both drug except SDS showed increase of CFX intensity but caused decrease in PLN intensity. So, it was preferred that the work was carried out without adding any organized media (Fig. 8).

### Comparison between analytical performance of the proposed approach and other published procedures

By comparing the evolved technique with reported spectrophotometric [36] and high-performance liquid chromatographic [37] techniques, it was found that proposed method was simple, rapid and superior to other reported ones in term of sensitivity as its LOD was smaller than others. Moreover, it was the greenest method since green solvent (water) was used during whole study and its greenness was confirmed by four assessment tools as shown in (Table 1).

**Table 1:** Analytical performance of the suggested technique and other reported methods

Reference	Range (µg ml^−1^)		LOD (µg ml^−1^)		Technique	Assessment of greenness	Application
	PLN	CFX	PLN	CFX			
The proposed method	0.02–0.5	0.5–5.0	0.004	0.103	The assay principle based on first-order synchronous spectrofluorometric scan using Δ λ = 40 nm in which PLN peak amplitudes were recorded at 283.4 nm, meanwhile CFX was measured at 326.2 nm in the same scans	The method greenness was assessed and confirmed by four assessment tools; GAPI, NEMI, AGREE and Analytical Eco Scale Score	PLN and CFX in their pure forms, single and combined dosage forms
[36]	5.0–30.0	1.0‐12.0	1.16	0.110	Dual wavelength spectrophotometric simultaneous determination of PLN and CFX. The wavelengths selected for assay of CFX were 272.09 nm and 305.85 nm, and for PLN were 256.80 nm and 282.96 nm using 0.1 M NaOH as solvent	No assessment	PLN and CFX in pure forms and combined dosage forms only
[37]	5.0–30.0	150–900	0.220	2.19	A stability-indicating reversed-phase HPLC method was developed for simultaneous assay of PLN and CFX using C18 column and a mobile phase consisting of Water: Acetonitrile: Triethylamine (85: 15: 0.1, v/v/v), pH 3	No assessment	PLN and CFX in pure forms and combined dosage forms.only

### Validation parameters

Following International Council for Harmonization (ICH) recommendations [38], the method validity was investigated regarding; range, linearity, limits of detection and quantification, precision, accuracy and selectivity.

#### Range and linearity

Calibration curves showed linear relationship between ^1^D amplitudes and corresponding concentration of each drug within the ranges 0.02–0.5 µg/mL and 0.5–5.0 µg/mL for PLN and CFX, respectively. The derived regression equations are:

$${\mathbf{For}} \, {\mathbf{PLN}}:^{{1}} {\text{D}}\, = \,{59}.{\text{477 C}}\, + \,0.{19}0{\text{ at 273}}.0{\text{ nm }}({\text{r}}\, = \,0.{9999})$$

$${\mathbf{For}} \, {\mathbf{CFX}}:^{{1}} {\text{D}}\, = \,{1}0.{\text{147 C}}{-\!\!-}{1}.0{\text{82 at 326}}.{\text{2 nm }}({\text{r}}\, = \,0.{9998})$$

where ^**1**^**D** is the peak amplitude of first derivative synchronous fluorescence spectra, **C** is the drug concentration (µg/mL) and **r** is correlation coefficient. The statistical data confirming the suggested method linearity are abridged in (Table 2).

**Table 2:** Analytical performance of the suggested first derivative synchronous method

Parameters	CFX	PLN
Wavelength difference (Δ λ)	= 40 nm	
Linear range (μg/mL)	0.5–5.0	0.02–0.5
Intercept (*a*)	− 1.082	0.190
Slope (*b*)	10.147	59.477
Correlation coefficient (*r*)	0.9998	0.9999
Residuals S.D (S_*y/x*_)	0.408	0.131
Intercept S.D (S_*a*_)	0.318	0.074
Slope S.D (S_*b*_)	0.105	0.326
Percentage relative standard deviation (% RSD)	1.269	1.403
Percentage relative error (*%* Error)	0.518	0.575
Limit of detection, LOD (μg/mL)	0.103	0.004
Limit of quantitation, LOQ (μg/mL)	0.313	0.012
Number of experiments (n)	6	6

#### Limit of detection (LOD) and limit of quantitation (LOQ)

LOD and LOQ were computed mathematically (Table 2) by the equations stated by ICH Guidelines [38]:

$${\mathbf{LOD}}\, = \,({3}.{\text{3 S}}_{{\text{a}}} /{\text{b}})$$

$${\mathbf{LOQ}}\, = \,({1}0{\text{ S}}_{{\text{a}}} /{\text{b}})$$

where **S**_**a**_ is the intercept standard deviation and** b** is the slope of calibration curve.

#### Accuracy and precision

The proposed method was evaluated regarding accuracy through its application in the estimation of PLN and CFX in their pure forms and comparing the results with those obtained by comparison method [36] as represented in (Table 3).

**Table 3:** Data for the estimation of CFX and PLN in their pure form by the suggested first derivative synchronous method

Studied drugs	Conc taken (µg /mL)	% Found ^a^	Comparison method [36] % Found
CFX	0.5	100.20	97.85
	1.0	99.30	100.63
	2.0	101.50	101.75
	3.0	98.10	98.99
	4.0	101.25	
	5.0	99.68	
Mean ± SD	100.01 ± 1.27		99.81 ± 1.73
t	0.21*		
F	1.58*		
PLN	0.02	100.00	98.70
	0.05	102.00	102.04
	0.07	101.43	98.62
	0.1	101.00	100.35
	0.2	98.00	
	0.5	100.20	
Mean ± SD	100.44 ± 1.41		99.93 ± 1.62
t	0.53*		
F	1.32*		

^a^ The average of 3 separate results

^*^ The tabulated** t **and **F** values were **2.31, 5.41,** respectively at *P* = 0.05 [39]

The comparison method recommended dual wavelength spectrophotometric determination of both drugs and the wavelengths selected for assay of CFX were 272.09 nm and 305.85 nm, while, the wavelengths selected for the assay of PLN were 256.80 nm and 282.96 nm by using 0.1 M NaOH as solvent. Statistical analysis of the data using Student’s t-test and variance ratio F-test [39] showed no remarkable discrepancy between two methods confirming accuracy of the proposed method.

**Intra day precision:** It was assessed by assay of three concentrations of each drug in pure forms three sequencing times on the same day and **Inter day precision** was confirmed through repeated assay in 3 successive days.

SD and % RSD small values as in (Table 4) accentuated high precision of the evolved method.

**Table 4:** Precision data of the proposed first derivative synchronous method

Drug	Conc. (µg /mL)	Inter-day precision			Intra-day precision		
		Mean ± SD	% RSD	% Error	Mean ± SD	% RSD	% Error
CFX	0.5	100.16 ± 0.98	0.98	0.56	100.22 ± 1.08	1.08	0.62
	3.0	99.36 ± 1.55	1.56	0.90	99.57 ± 1.48	1.48	0.85
	5.0	99.95 ± 1.18	1.19	0.68	99.68 ± 0.98	0.98	0.57
PLN	0.05	100.42 ± 1.35	1.35	0.78	99.86 ± 1.21	1.21	0.70
	0.1	99.64 ± 1.61	1.62	0.93	99.64 ± 1.28	1.29	0.74
	0.5	100.35 ± 1.18	1.18	0.68	99.79 ± 1.08	1.08	0.62

#### Selectivity

This proposed spectrofluorimetric method showed that each drug can be analyzed in the mixture with no interference from the other drug confirming method specificity and its ability for resolving a mixture of the two studied drugs (Table 5). The suggested method selectivity was confirmed by determination of the two drugs in their single and combined eye drops with high % found with no interference from common additives as shown in Tables 6 and 7.

**Table 5:** Data for the assay of CFX and PLN in their synthetic mixtures by the suggested first derivative synchronous method and comparison one

Parameters	Proposed method				Comparison Method [36]	
	Conc. taken (µg/mL)		% Found ^a^		% Found ^a^	
	CFX	PLN	CFX	PLN	CFX	PLN
	0.6	0.02	99.89	100.03	100.21	101.42
	1.5	0.05	101.05	99.53	101.25	99.28
	2.1	0.07	98.92	98.71	99.51	100.47
	3.0	0.1	100.45	99.36	102.04	101.78
Mean			100.08	99.41	100.75	100.74
± S.D			0.90	0.54	1.11	1.12
%RSD			0.90	0.54		
%Error			0.45	0.27		
t			0.93*	2.14*		
F			1.5*	4.24*		

^a^ The average of 3 separate results

^*^ The tabulated** t **and **F** values were **2.45, 9.28,** respectively at *P* = 0.05 [39]

**Table 6:** Assay of CFX and PLN in their single commercial eye drops by the suggested first derivative synchronous method

Parameter	Conc taken (µg /mL)	% Found ^a^	Comparison method [36] % Found ^a^
Ciprocin® eye drops (0.3% CFX)	0.5	100.16	100.21
	1.0	99.35	99.69
	2.0	98.95	101.95
	3.0	102.09	98.90
Mean ± SD	100.14 ± 1.39		100.19 ± 1.29
t	0.04		
F	1.17		
Phenylmedrin® eye drops (2.5% PLN)	0.02	98.35	101.43
	0.05	99.53	102.14
	0.07	100.16	100.48
	0.1	101.89	99.64
Mean ± SD	99.98 ± 1.48		100.92 ± 1.09
t	1.03*		
F	1.82*		

^**a**^ The average of three separate results

^*^ The tabulated **t** and **F** values are** 2.45** and **9.28** respectively at *p* = 0.05 [39]

**Table 7:** Data for the assessment of CFX and PLN in their prepared combined eye drops using this first derivative synchronous and comparison methods

Parameters	Suggested method				Comparison method [36]	
	Conc. taken (µg/mL)		% Found ^a^		% Found ^a^	
	CFX	PLN	CFX	PLN	CFX	PLN
Prepared eye drops (0.3%CFX + 0.01%PLN)	0.6	0.02	101.53	101.71	101.25	98.57
	1.5	0.05	99.08	98.18	99.16	100.71
	2.1	0.07	100.80	98.71	99.51	101.42
	3.0	0.1	99.79	101.04	100.73	99.64
Mean			100.30	99.91	100.16	100.08
± S.D			1.08	1.72	0.98	1.25
%RSD			1.07	1.72		
%Error			0.54	0.86		
t			0.19*	0.16*		
**F**			1.20*	1.90*		

^a^ The average of three separate results

^*^ The tabulated **t** and **F** values are **2.45** and **9.28** respectively at p = 0.05 [39]

^*^ The tabulated t and F values are 2.45 and 9.28 respectively at p = 0.05 [39]

### Application

#### Assay of CFX / PLN synthetic mixtures

This derivative synchronous spectrofluorimetric technique was applied for synchronized assay of CFX and PLN in synthetic mixtures in their ratio (30:1) as represented in (Fig. 4). By using the derived regression equations, each drug percent found in their synthetic mixture could be determined.

#### Assay of CFX and PLN in their single commercial eye drops

The proposed technique was applied for analysis of CFX and PLN in their single ophthalmic dosage forms and the attained results were compared with those of comparison method [36] as presented in Table 6. These data were statistically assessed utilizing Student's t‐test and variance ratio F ‐test [39] and it was found that there were no remarkable differences between the two procedures.

#### Assay of CFX and PLN in their prepared co-formulated eye drops

The proposed technique was perfectly applied for simultaneous assay of the two studied drugs in their combined dosage form to authorize its efficient application in quality control laboratories. The results presented in Table 7 were close to the comparison method results [36]. Furthermore, Student's t‐test and variance ratio F ‐test [39] deduced no remarkable differences between the two methods regarding accuracy and precision.

## Evaluation of the proposed technique greenness

Nowadays green analytical chemistry attracts more attention so it is important to assess the ecological impact of the evolved method. Various analytical matrices such as Green Analytical Procedure Index (GAPI), analytical Eco-scale, National Environmental Methods Index (NEMI) and Analytical Greenness (AGREE) were conducted (Table 8).

**Table 8:** Evaluation results of the greenness of suggested technique

(1) Green Analytical Procedure Index (GAPI)	

(2) NEMI pictogram	(3) AGREE pictogram
	
(4) Analytical Eco-Scale Score	
Item	Penalty points
1-Reagent Water	0
2-Spectrofluorimeter	0
3-Occupational hazard	0
4-Waste	3
Total penalty points	3
Analytical Eco- Scale score	97

GAPI is a certain symbol with five pentagrams which can be used for not only providing an immediately perceptible perspective to the analyst but also gives exhaustive information on each step of an analytical methodology from sample collection, sample preparation, solvents, reagents and instrumentation to final analysis [40].

Analytical Eco-Scale relies on the concept that the total penalty points of the parameters of the method (energy, amount of reagent, hazard and waste) can be calculated then subtracted from 100 to give value determines its greenness [41]. It was found that the proposed method score equals 97, pointing out the excellent degree of the proposed method greenness.

NEMI is a qualitative method based on using a certain circular pictogram which consists of four quadrants [42]: Each of them corresponding to a certain parameter. By applying it to the developed method, it was found that the four criteria of greenness were fulfilled and represented by four green parts. The NEMI criteria include: persistent, bio accumulative, toxic (PBT), hazardous, corrosive and waste. Although it is a feasible readable method, it does not demonstrate any quantitative determination.

AGREE is a graph that resembles a clock and has twelve sectors around its perimeter [43] One of the twelve principles of Green Analytical Chemistry (GAC) is represented by each sector. A red, orange, and green scale is used to indicate how well the method performs in relation to each of the GAC principles. The overall color in the center of the graph, which has a score ranging from 0 to 1, represents the method's overall performance. The suggested approach, as shown in Table 8, has a score of 0.82 and a green core, indicating that the evolved method is green.

The greenness of the proposed method was compared with that of the other previously reported methods for simultaneous determination of CFX and PLN [35, 36]. It was found that suggested method was more green and safe as shown in Table 9.

**Table 9:** Greenness of the suggested technique and other reported methods

	Proposed method	Reported spectrophotometric method [36]	Reported HPLC method [37]
GAPI			
NEMI			
AGREE			
Eco-Scale	Item Penalty points 1-Reagent Water 0 2-Spectrofluorimeter 0 3-Occupational hazard 0 4-Waste 3 Total penalty points 3 Eco- Scale score 97	Item Penalty points 1-Reagent NaOH 1 2-UV- Vis Spectrometer 0 3-Occupational hazard 0 4-Waste 3 Total penalty points 4 Eco- Scale score 96	Item Penalty points 1-Reagent Methanol 12 Acetonitrille 8 Phosphate buffer 0 2-HPLC–UV 1 3-Occupational hazard 0 4-Waste 3 Total penalty points 24 Eco- Scale score 76

## Conclusion

A simple, green, sensitive and rapid first derivative synchronous spectrofluorometric technique has been developed for concurrent assay of CFX and PLN in pure forms, single and combined dosage forms. The evolved technique was fully validated according to ICH Guidelines. Moreover, the greenness was investigated using four different matrices; GAPI, NEMI, AGREE and Analytical Eco-Scale and the obtained results confirmed eco-friendliness of the proposed method. Consequently, these properties authorize application of this method in quality control of the studied drugs.

## Data Availability

The datasets used and/or analyzed during the current study are available from the corresponding author on reasonable request.
